# It is time to rethink tactics in the fight against malaria

**DOI:** 10.1186/1475-2875-12-140

**Published:** 2013-04-24

**Authors:** Laurence Slutsker, S Patrick Kachur

**Affiliations:** 1 Division of Parasitic Diseases and Malaria, Centers for Disease Control and Prevention, 1600 Clifton Road, MS A-06, Atlanta, GA, 30333, USA

## Abstract

April 25 marks World Malaria Day, an opportunity for those who work to defeat the illness, to review progress and renew commitments. After a decade of steady success, this year’s commemoration of the date is also an opportunity to reconsider current approaches and assess the state of the science needed to keep pace in the global effort to combat malaria.

## Commentary

Malaria control efforts are one of the biggest success stories in global health in the last decade. With substantially increased funding for four key tools—insecticide-treated bed nets, effective treatment drugs, preventive treatment for pregnant women, indoor spraying of homes with insecticides — all of them grounded in strong science, more than a million lives have been saved. The number of people who become sick or die from malaria has decreased by 25% globally and by 33% in the hardest-hit region, sub-Saharan Africa. Some countries supported by the President’s Malaria Initiative (PMI), the U.S. government’s effort focusing largely on sub-Saharan Africa, have cut their malaria burden in half.

These successes are encouraging, to say the least. However, malaria still kills approximately 660,000 people each year, mostly children. World Malaria Day, April 25th, and its theme “Invest in the future. Defeat malaria,” remind the global community of the ultimate goal and the need to keep fighting.

The challenge now is to know where people are being infected and tailor the tools that programmes are using so that they are most effective. Reaching ever larger numbers of people in countries with life-saving malaria interventions has saved lives; in some areas, it has changed the malaria environment itself. Some countries with once uniformly high levels of malaria transmission may now have a varied malaria landscape: low transmission in some areas, malaria “hot spots” in others. Other countries that used to have low transmission can now begin moving toward elimination of malaria. In some countries with changed malaria landscapes, national malaria control programmes and partners are now beginning to adjust strategies.

Take Zambia, for example. A PMI participant country, Zambia has scaled up the four key tools within its borders. As a result, the number of malaria infections in parts of the country had plummeted. Zambia then established intensive surveillance in a part of the country where malaria infection was generally low. Mobile phone technology began to be used in health centres to report malaria cases, in real time to district-level managers, who then make sure nets and drugs will be where they are needed. In areas with low malaria infection, health centres determine whether patients that test positive for malaria were likely infected locally and if so, visit their homes to test family and treat those who tested positive. This intervention, called “reactive case detection,” is one way to target efforts to reach areas and individuals most in need, be more efficient, and make progress toward decreasing transmission, and thus, the risk of malaria infection.

Malaria is in retreat in many areas, but it remains a dangerous and resilient foe. CDC continues to invest its scientific expertise as a leader in global malaria control activities. Continued success in the fight against malaria requires persistence, cunning, and, most of all, knowledge. Once it is known where people are being infected with malaria, approaches can be adjusted to match the need. With this, national malaria control programmes and their development partners can adapt to changing realities and continue to save lives and improve people’s health. And the essential tools for prevention and treatment can be put to use where they will do the most good. Only then is there a fighting chance to defeat this long-time foe.

